# The Prevalence and Impact of Allergic Rhinitis on Asthma Exacerbations in Asthmatic Adult Patients in the Riyadh Region of Saudi Arabia: A Cross-Sectional Study

**DOI:** 10.7759/cureus.32324

**Published:** 2022-12-08

**Authors:** Turki Bin Mahfouz, Shaima A Banjar, Raghad A Assiri, Ghada A Alshehri, Faris Binyousef

**Affiliations:** 1 Otolaryngology - Head and Neck Surgery, Imam Mohammad Ibn Saud Islamic University, Riyadh, SAU; 2 Medicine and Surgery, College of Medicine, Imam Mohammad Ibn Saud Islamic University, Riyadh, SAU

**Keywords:** asthma, allergic rhinitis, general medicine, general practice, allergic rhinitis and its impact on asthma, asthma control, allergic rhinitis (ar), asthma severity

## Abstract

Objectives: Allergic rhinitis (AR) and asthma are highly prevalent conditions known to occur concomitantly. However, observational, cross-sectional studies in Saudi Arabia assessing the frequency and severity of rhinitis in asthmatics adults using questionnaires based on guidelines are unavailable. Therefore, this study attempted to investigate this side and evaluate the role of triggers, symptoms, and family history or history of AR on asthma control levels.

Methods: From April 2nd to September 18th, 2021, this observational cross-sectional study was conducted through an online self-administrated questionnaire that was distributed electronically on social media through the SurveyMonkey website (Momentive Inc., Waterford, NY). The study targeted asthmatic adult patients residing in Riyadh city in Saudi Arabia.

Results: Overall, 187 questionnaires were analyzed. In this study, the frequency of AR in asthmatic patients was 75.5% (95% CI: 74.87-75.4%). Of those, AR was intermittent mild for 15.0%, intermittent moderate to severe for 43.9%, mild persistent for 2.14%, persistent moderate to severe for 14.4%, and 24.6% of patients were without AR. A significant association was observed between asthma control level and the severity of AR (P < 0.001). Moderate to severe persistent AR was more prevalent in patients with uncontrolled asthma (40.6%) than patients with partially controlled (25%) or controlled asthma (2.7%).

Conclusions: This study suggested that AR was related to more severe asthma and more difficulty in controlling asthma. The frequency significantly increased with the severity of asthma.

## Introduction

The overlap between asthma and allergic rhinitis (AR) was recognized years ago. The immune system has the greatest part in the pathophysiology of the two diseases. Later studies identified that asthma severity could provoke an inflammatory response in the upper respiratory tract, enhancing the likelihood of AR occurrence [1]. Asthma affects approximately 235 million of the world’s population [2]. In Saudi Arabia, asthma is one of the most common chronic respiratory diseases affecting more than 2 million Saudis [2]. According to the Saudi Initiative for Asthma (SINA 2016), based on research completed over the last three decades, the general incidence of asthma in children varies from 8% to 25% [2]. AR is prevalent in Saudi Arabia; a study conducted in Al-Ahsa city concluded that 76% of the population had AR [3]. Thus, the shortfall of studies about the bond between asthma and AR in the last decade encourages researchers to investigate that link in the capital of Saudi Arabia.

Asthma is a common disease worldwide, and it is considered a chronic respiratory disease. According to the World Health Organization, it is characterized by recurrent attacks of shortness of breath, coughing, and wheezing. Nevertheless, asthma severity varies from one person to another [4]. However, AR is also considered a chronic respiratory disease, described as an inflammation of the nasal mucosa resulting from exposure to allergen triggers such as trees, weed pollens, and house dust mites [5]. AR has four main criteria: rhinorrhea, nasal itching, nasal congestion, and sneezing [6]. The number of days per week and consecutive weeks per year when a patient is symptomatic should be evaluated to establish the AR severity, according to the Allergic Rhinitis and its Impact on Asthma (ARIA) guidelines. AR is subcategorized into intermittent AR (symptoms < four days/week or for < four weeks) and persistent AR (symptoms > four days/week or for > four weeks) and is further characterized according to severity as mild or moderate/severe [7]. However, most patients often express various shades of impairments, including non-nasal symptoms. Yet the lack of insight and studies regarding this aspect in Saudi Arabia is observed.

This study attempted to investigate the prevalence and impact of AR severity on asthma exacerbations and assess the role of triggers, symptoms, and AR history or family history on asthma control level using the score of self-administered short-form allergic rhinitis (SFAR) questionnaire and ARIA guidelines.

## Materials and methods

Study design

It is an observational, descriptive, cross-sectional study derived from a self-administered questionnaire of adult residents in the Riyadh region of Saudi Arabia.

Study population, sample size, and technique

This study targeted adult patients with asthma in the Riyadh region, with randomly selected participants enrolled through a questionnaire distributed online. A previous study concluded that AR prevalence in patients with asthma is 78% [6]. Thus, the sample size was suggested to be 150 patients based on the sample size calculation formula: sample size = [Z2p (1 − p)]/C2 with a 95% CI and a margin of error of 3%. In this study, the sample size was 141 patients with a 95% CI and a margin of error of 3.4%.

Population selection

This research investigated the known association between asthma and AR. The inclusion criteria included the following: Riyadh region residents aged 16-66 years, previously diagnosed clinically with asthma for more than six months, and agreed to participate in the study. Furthermore, the exclusion criteria of participants included the following: anyone who had chronic obstructive pulmonary disease (COPD), pulmonary fibrosis or bronchitis, or pneumonia, had been diagnosed with coronavirus disease 2019 (COVID-19) for less than six months, disagreed to participate in the study, not living in Riyadh city or any of its provinces, and aged < 16 years old.

Data collection and questionnaire

Data collection began from April 2, 2021, to September 18, 2021, through an online self-administrated questionnaire distributed electronically on social media through the SurveyMonkey website (Momentive Inc., Waterford, NY). All participants who chose to disagree with research participation at the beginning of the questionnaire immediately went to the submission page to submit their responses.

The questionnaire contained four major sections: participants’ demographic, the SFAR questionnaire, ARIA guidelines, and the Asthma Control Test (ACT) questionnaire.

Population demographics included age, region, gender, education, body mass index (BMI), and smoking status. SFAR is a simple, valid diagnostic tool in AR with sensitivity and specificity rates of 94.8% and 95.1%, respectively [8]. SFAR evaluates eight questions associated with AR symptoms, time of occurrence during the year, triggers, allergy tests, and personal and family allergy histories [9]. Patients with an SFAR score of ≥7 are considered to have clinical manifestations of AR, whereas patients with an SFAR score of <7 are deemed to be free of AR [7]. SFAR provides a precise categorization of the population as either AR or AR-free, which established a solid step to further categorize the AR and associate it with asthma severity according to the ARIA guidelines. This research used the Arabic version of the SFAR questionnaire, which was validated by research conducted in Saudi Arabia to estimate the prevalence of AR [10].

The ARIA guidelines were used to categorize the degrees of AR-related asthma. ARIA arranges the severity of AR (intermittent/persistent and mild/moderate to severe) based on the duration of the symptoms, either intermittent or persistent course, the intensity of AR severity, and its impact on the distribution of the quality of life of patients with asthma [5]. Intermittent AR is present when patients experience the symptoms for less than four days per week or less than four weeks. Persistent AR is present when patients experience the symptoms for more than four days per week or more than four weeks. Mild is described as regular sleep, no impairment of daily activity, normal work and school, and no troublesome symptoms. Moderate-to-severe AR includes abnormal sleep, impairment of daily exercise, impairment in work and school activities, and troublesome symptoms. The severity is classified based on “yes” or “no” answers to the previous four items in question form. Questions related to ARIA guidelines were taken from the previously validated ARIA guidelines [10]. The ARIA questionnaire was translated into Arabic, the official language of Saudi Arabia, by a bilingual Saudi physician. The questionnaire was back-translated into English by another translator who does not know the English version. Forward translation into Arabic was conducted. Subsequently, the Arabic version was used in a pilot test for understanding in a small patient group before it was used.

The ACT questionnaire is a self-administered instrument for identifying patients with poorly managed asthma. The ACT questionnaire comprises five Likert scale items ranging from one to five on a scale of one to five describing night and daytime symptoms and activity limits. The overall score for the ACT is estimated by summing the scores for the five items with a possible score ranging from 5 (poor control of asthma) to 25 (complete control of asthma). Higher scores reflect better asthma control [11]. ACT cut-off points for uncontrolled and partly controlled asthma were ≤19 and ≤22, respectively [12]. Respondents whose asthma was found as controlled or completely controlled were grouped during the analysis. The ACT has been validated on smoker participants [13]. Therefore, in this research, an ACT-validated Arabic edition was used [14].

Statistical analysis

Responses were gathered and transferred to an Excel spreadsheet (Microsoft Corporation, Redmond, WA) for additional examination. R version 3.6.3 (R Foundation for Statistical Computing, Vienna, Austria) was used for statistical analysis. The distribution of categorical variables was summarized using counts and percentages. The mean ± standard deviation was used for continuous variables. The chi-square test of independence was used to assess the association between categorical variables. Unpaired t-tests and one-way ANOVA were used to compare the distribution of continuous variables between groups with two and more than two levels, respectively. For one-way ANOVA, post hoc comparisons were conducted using an unpaired t-test with correction for false discovery rate. Hypothesis testing was performed at a 5% level of significance.

Ethical approval

The research obtained personal data and patient information. Participation was voluntary, and informed consent was obtained from all participants before they enrolled in this study. All participants were provided with sufficient information regarding the study’s aims. Participants’ data remained confidential and were used for research purposes only. A scientific committee and institutional review board at Imam Mohammed Ibn Saud Islamic University were set up to verify the project’s scientific quality, the relevance of the objectives and methodology, and control the progress of the study. This study was conducted following the Helsinki Declaration and was approved by local legal authorities, including the Internal Review Board (IRB) for Ethics in Research on Living Creatures at the University of Imam Muhammad Bin Saud Islamic University​ (approval number: 135-2021).

## Results

**Figure 1:**
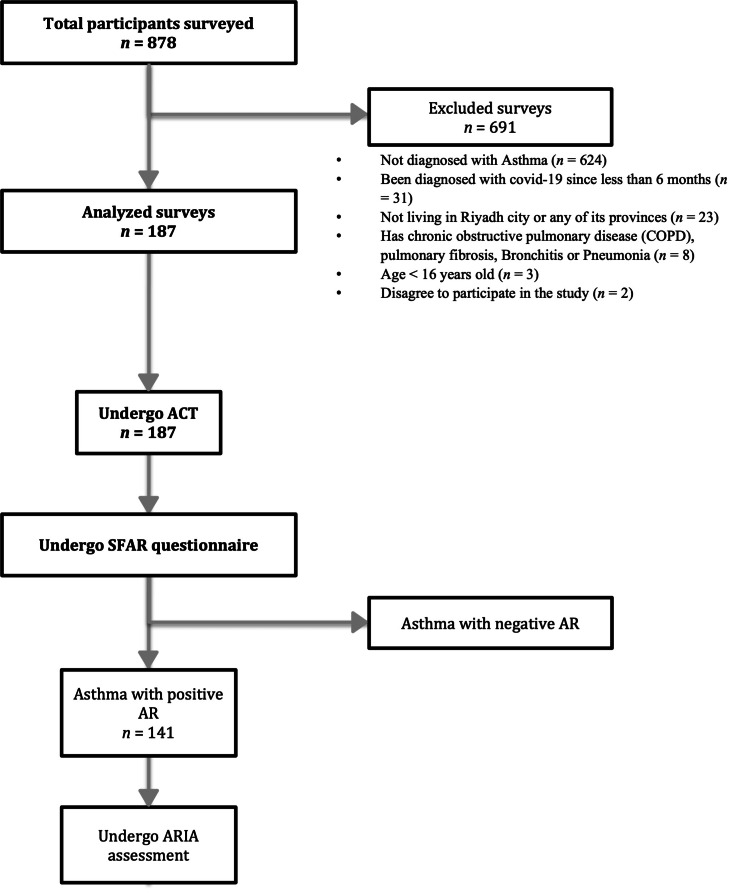
Flow diagram of the study AR: allergic rhinitis; ACT: Asthma Control Test; ARIA: Allergic Rhinitis and its Impact on Asthma; SFAR: short-form allergic rhinitis.

Overall, 878 respondents completed the study questionnaire between April and September 2021. Altogether, 691 participants (78.7%) were excluded from the analyzed population due to incompatibility with the inclusion criteria. Incompatibilities included not being diagnosed with asthma (n = 624), being diagnosed with COVID-19 in less than six months (n = 31), not living in Riyadh city or any of its provinces (n = 23), having COPD, pulmonary fibrosis, bronchitis, or pneumonia (n = 8), age < 16 years old (n = 3), and disagree to participate in the study (n = 2). Overall, the study analyzed 811 questionnaires. Incomplete questionnaires were not allowed to be submitted as responses. The study flow is shown in Figure 1.

Respondents’ characteristics

Overall, 187 questionnaires were analyzed. Of these, 96.3% were living in Riyadh city, and 3.7% were living in one of the Riyadh provinces. Males and females represented 55.1% and 44.9% of the study sample, respectively. Respondents aged 16-31 years old represented 70.6% of the study sample, whereas respondents aged 32-47 and 48-63 years represented 18.7% and 10.7% of the study sample, respectively. Notably, no respondents were aged 64-66 years. Most respondents had a normal BMI (54%), and overweight and obese respondents represented 21.4% and 20.3% of the study sample, respectively. Most respondents were non-smokers (84.0%), and 12.3% were current smokers. Most respondents had AR (75.4%). Furthermore, 71.1% of asthmatic patients have been diagnosed for more than five years, 19.8% for less than two years or more than six months, and 9.09% for less than five years and more than two years. The demographic characteristics of the patients are presented in Table 1, and the main results are shown in Table 2.

**Table 1:** Demographic and clinical characteristics of respondents

	Overall population, n (%) (N = 187)	
		Sex		
Female	103 (55.1%)	
Male	80 (44.9%)	
Age		
16-31	132 (70.6%)	
32-47	35 (18.7%)	
48-63	20 (10.7%)	
BMI		
Underweight	8 (4.3%)	
Normal	101 (54.0%)	
Overweight	40 (21.4%)	
Obese	38 (20.3%)	
History of eczema, allergic rhinitis		
No	116 (62.0%)	
Yes	71 (38.0%)	
Smoking state		
Never smoked	157 (84.0%)	
Ex-smoker	7 (3.74%)	
Current smoker	23 (12.3%)	
Trigging factors		
Pollen	32 (17.1%)	
House dust mites	38 (20.3%)	
Dust	77 (41.2%)	
Animals	27 (14.4%)	
Fungi & mold	0 (0%)	
Other	0 (0%)	
Asthma diagnosis duration		
Yes, less than five years and more than two years	17 (9.1%)	
Yes, more than five years	133 (71.1%)	
Yes, recently two years or more than six months	37 (19.8%)	
Treatment		
Intranasal corticosteroids	48 (25.7%)	
Antihistamine	30 (16.0%)	
Leukotriene receptor antagonists (LTRA)	0 (0%)	
Do not use any	114 (61.0%)	

**Table 2:** Association between demographic characteristics, asthma control, and presence of allergic rhinitis (AR) Counts and percentages were used to summarize the variables

	Overall population, n (%) (N = 187)	Controlled, n (%)		Partially controlled, n (%)		Uncontrolled, n (%)	
		-AR, n (%) (N = 38)	+AR, n (%) (N = 73)	-AR, n (%) (N = 3)	+AR, n (%) (N = 41)	-AR, n (%) (N = 5)	+AR, n (%) (N = 27)
History of eczema, AR							
No	116 (62.0%)	32 (84.2%)	35 (47.9%)	0 (0.00%)	23 (56.1%)	5 (100%)	21 (77.8%)
Yes	71 (38.0%)	6 (15.8%)	38 (52.1%)	3 (100%)	18 (43.9%)	0 (0.00%)	6 (22.2%)
BMI							
Underweight	8 (4.28%)	0 (0.00%)	5 (6.85%)	0 (0.00%)	3 (7.32%)	0 (0.00%)	0 (0.00%)
Normal	101 (54.0%)	25 (65.8%)	42 (57.5%)	3 (100%)	19 (46.3%)	3 (60.0%)	9 (33.3%)
Overweight	40 (21.4%)	8 (21.1%)	8 (11.0%)	0 (0.00%)	11 (26.8%)	2 (40.0%)	11 (40.7%)
Obese	38 (20.3%)	5 (13.2%)	18 (24.7%)	0 (0.00%)	8 (19.5%)	0 (0.00%)	7 (25.9%)
Smoking state							
Never smoked	157 (84.0%)	33 (86.8%)	68 (93.2%)	3 (100%)	34 (82.9%)	3 (60.0%)	16 (59.3%)
Ex-smoker	7 (3.74%)	2 (5.26%)	0 (0.00%)	0 (0.00%)	0 (0.00%)	2 (40.0%)	3 (11.1%)
Current smoker	23 (12.3%)	3 (7.89%)	5 (6.85%)	0 (0.00%)	7 (17.1%)	0 (0.00%)	8 (29.6%)
Trigging factors							
Pollen	32 (17.1%)	3 (7.89%)	21 (28.8%)	0 (0.00%)	8 (19.5%)	0 (0.00%)	0 (0.00%)
House dust mites	38 (20.3%)	6 (15.8%)	20 (27.4%)	0 (0.00%)	6 (14.6%)	0 (0.00%)	6 (22.2%)
Dust	77 (41.2%)	10 (26.3%)	35 (47.9%)	0 (0.00%)	20 (48.8%)	0 (0.00%)	12 (44.4%)
Animals	27 (14.4%)	8 (21.1%)	10 (13.7%)	0 (0.00%)	3 (7.32%)	0 (0.00%)	6 (22.2%)
Fungi & mold	0 (0%)	0 (0%)	0 (0%)	0 (0.00%)	0 (0.00%)	0 (0.00%)	0 (0.00%)
Other	0 (0%)	0 (0%)	0 (0%)	0 (0%)	0 (0%)	0 (0%)	0 (0%)
Treatment							
Intranasalcorticosteroids	48 (25.7%)	0 (0.00%)	18 (24.7%)	0 (0.00%)	17 (41.5%)	0 (0.00%)	13 (48.1%)
Antihistamine	30 (16.0%)	2 (5.26%)	10 (13.7%)	0 (0.00%)	10 (24.4%)	0 (0.00%)	8 (29.6%)
Leukotriene receptor antagonists (LTRA)	0 (0%)	0 (0.00%)	0 (0.00%)	0 (0.00%)	0 (0.00%)	0 (0.00%)	0 (0.00%)
Do not use any	114 (61.0%)	36 (94.7%)	47 (64.4%)	3 (100%)	14 (34.1%)	5 (100%)	9 (33.3%)

Asthma control and AR severity

**Figure 2:**
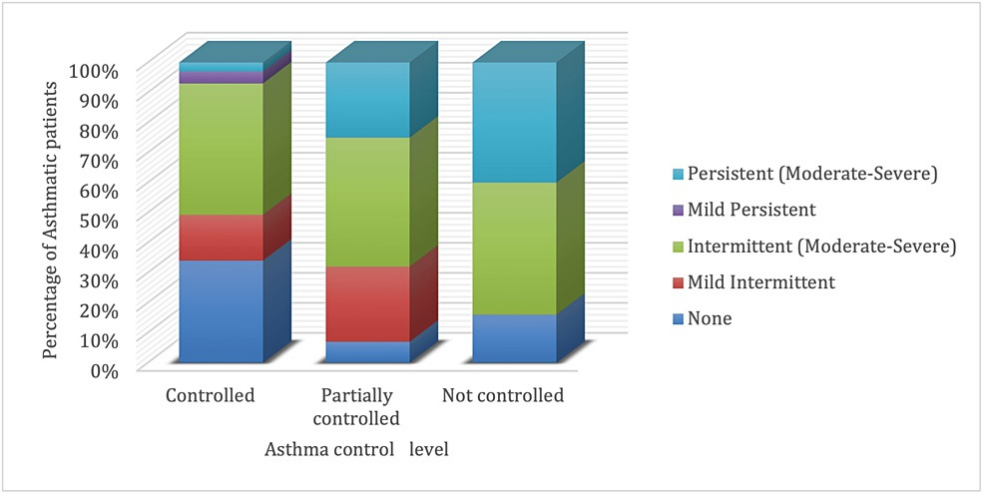
Distribution of allergic rhinitis severity among different asthma control levels Asthma control level was controlled in 59.4% of patients, partially controlled in 23.5%, and not controlled at all in 17.1% of patients.

The percentages of affirmative responses to the rhinitis questionnaire are presented according to the severity of asthma in Table 2. A statistically significant association was observed between asthma control level and the grade of AR as assessed by the ACT and ARIA questionnaires, respectively (P < 0.001). Moderate to severe persistent AR was more prevalent in patients with uncontrolled asthma (40.6%) than patients with partially controlled (25%) or controlled asthma (2.7%). The results imply a positive association between asthma and AR control. Moderate to severe intermittent AR was insignificantly different between the three groups (P = 0.994). Additionally, the proportion of patients with no AR was significantly higher in patients with controlled asthma than in patients with uncontrolled or partially controlled asthma (P = 0.001) (Table 3 and Figure 2).

**Table 3:** Asthma control and AR severity Counts and percentages were used to summarize the study variables. Statistical analysis was performed using the chi-square test of independence. P-values for the overall association. AR: allergic rhinitis; ARIA: Allergic Rhinitis and its Impact on Asthma.

	All	Not controlled at all	Partially controlled	Controlled	P overall
	N = 187	N = 32	N = 44	N = 111	
ARIA result					<0.001
None	46 (24.6%)	5 (15.6%)	3 (6.82%)	38 (34.2%)	0.001
Intermittent mild	28 (15.0%)	0 (0.00%)	11 (25.0%)	17 (15.3%)	0.01
Intermittent (moderate-severe)	82 (43.9%)	14 (43.8%)	19 (43.2%)	49 (44.1%)	0.994
Mild persistent	4 (2.14%)	0 (0.00%)	0 (0.00%)	4 (3.60%)	0.025
Persistent (moderate-severe)	27 (14.4%)	13 (40.6%)	11 (25.0%)	3 (2.70%)	<0.001

Allergic rhinitis

The prevalence of sneezing and runny or blocked noses during the past 12 months was insignificantly different based on asthma control level (P = 0.195). Similarly, the prevalence of itchy-watery eyes (P = 0.148) and seasonal pattern of the nose problem (P = 0.136) were not significantly different between patients with controlled, partially controlled, and controlled asthma. Furthermore, sensitivity to triggering factors was significantly associated with asthma control level (P = 0.016). Patients with controlled and partially controlled asthma reported higher sensitivity to pollens than those with uncontrolled asthma, whereas sensitivity did not differ for the remaining allergens. Patients with partially controlled asthma were more likely to test positive for skin prick test (SPT) and immunoglobulin E (IgE) (13.6%) than those with controlled (0%) and uncontrolled asthma (0%), and the association was statistically significant (P < 0.001). The prevalence of eczema was not significantly different between the three groups (P = 0.159). Family history statistically correlated with asthma control (P = 0.031; Table 4).

**Table 4:** SFAR analysis and ACT For questions 1-3, all populations are the number of patients who answered the question with a yes. Counts and percentages were used to summarize the study variables. Statistical analysis was performed using the chi-square test of independence. AR: allergic rhinitis; SFAR: short-form allergic rhinitis; ACT: Asthma Control Test; SPT: skin prick test; IgE: immunoglobulin E.

	Asthma control level				
	Overall population, n (%)	Uncontrolled, n (%)	Partially controlled, n (%)	Controlled, n (%)	P-value
(1) Sneezing, runny nose, or blocked nose during the last 12 months?	70 (37.4%)	9 (28.1%)	21 (47.7%)	40 (36.0%)	0.195
(2) Nose problem accompanied by itchy-watery eyes?	59 (31.6%)	12 (37.5%)	18 (40.9%)	29 (26.1%)	0.148
(3) In which season did this nose problem occur? (Several answers allowed)					0.136
None	139 (74.3%)	26 (81.2%)	28 (63.6%)	85 (76.6%)	
Winter/autumn season	32 (17.1%)	6 (18.8%)	10 (22.7%)	16 (14.4%)	
Spring/summer season	16 (8.56%)	0 (0.00%)	6 (13.6%)	10 (9.01%)	
(4) What trigger factors provoke or increase your nose symptoms? (Several answers allowed)					
Pollen (from trees, flowers, and grass)	32 (17.1%)	0 (0.00%)	8 (18.2%)	24 (21.6%)	0.016
House dust mites, dust	38 (20.3%)	6 (18.8%)	6 (13.6%)	26 (23.4%)	0.382
Animals (especially cats and dogs)	27 (14.4%)	6 (18.8%)	3 (6.82%)	18 (16.2%)	0.232
Fungi and mold (inside or outside the house)	0 (0.00%)	0 (0.00%)	0 (0.00%)	0 (0.00%)	
(5) In which season did this nose problem occur? (Several answers allowed)					0.119
None	139 (74.3%)	26 (81.2%)	28 (63.6%)	85 (76.6%)	
Winter season	22 (11.8%)	6 (18.8%)	8 (18.2%)	8 (7.21%)	
Spring season	8 (4.28%)	0 (0.00%)	3 (6.82%)	5 (4.50%)	
Summer season	8 (4.28%)	0 (0.00%)	3 (6.82%)	5 (4.50%)	
Autumn/fall season	10 (5.35%)	0 (0.00%)	2 (4.55%)	8 (7.21%)	
(6) Tested for SPT and IgE					<0.001
Positive tested	6 (3.21%)	0 (0.00%)	6 (13.6%)	0 (0.00%)	
Not tested	181 (96.8%)	32 (100%)	38 (86.4%)	111 (100%)	
(7) Diagnosed suffered/suffering from eczema?					0.159
Yes	125 (66.8%)	17 (53.1%)	29 (65.9%)	79 (71.2%)	
No	62 (33.2%)	15 (46.9%)	15 (34.1%)	32 (28.8%)	
(8) Family member suffering from (asthma, eczema, AR)					0.098
No one in my family has these diseases	116 (62.0%)	26 (81.2%)	23 (52.3%)	67 (60.4%)	
Yes, father	21 (11.2%)	3 (9.38%)	5 (11.4%)	13 (11.7%)	
Yes, mother	15 (8.02%)	0 (0.00%)	3 (6.82%)	12 (10.8%)	
Yes, siblings	35 (18.7%)	3 (9.38%)	13 (29.5%)	19 (17.1%)	
(9) Family member suffering from (asthma, eczema, AR)					0.031
Yes	71 (38.0%)	6 (18.8%)	21 (47.7%)	44 (39.6%)	
No	116 (62.0%)	26 (81.2%)	23 (52.3%)	67 (60.4%)	

## Discussion

Respondents’ characteristics

According to Table 1, the number of female participants in this study constitutes the largest percentage compared to male participants. According to Skoner's study, 80% of occurrences of AR occur in adults between the ages of 20 and 40 years [15]. And this correlates with our result, which was most of the participants included were from the age group of 16-31 years. In the majority of instances, the patient’s BMI was normal. It is noteworthy that most uncontrolled and controlled patients did not smoke. As far as smoking status is concerned, no significant findings were discovered. It should be highlighted that prior studies did not thoroughly address the influence of obesity on AR. Nwaru et al. show the impact of asthma on adolescents and the link to high occurrences [16]. A statistically significant relationship (P = 0.031) was discovered between a family history of asthma and asthma control, with lower percentages for persons with partially managed or uncontrolled asthma. Additionally, Vázquez Nava et al. support the hypothesis that family atopy, active smoking, AR, and pollution increase the risk of asthma in adults [17]. Among all groups, dust remains the most frequent triggering factor, followed by mites and pollen. According to Badran et al., the SPT was positive in 77.8% of patients, primarily for grass pollen and dust mites [9].

Despite being managed or substantially controlled by AR, many patients continue to use intranasal corticosteroids for stability. Antihistamines were used in conjunction to minimize the intensity of symptoms in controlled patients with AR. However, a significant number of these individuals did not take any drugs.

Asthma control and AR severity

The severity of AR was signiﬁcantly related to poor asthma control in this study, and moderate to severe AR was high in patients who had not controlled their asthma at all. Furthermore, previous studies reported similar results that AR severity was related to asthma control. In Magnan’s study, severe AR was related to poor asthma control and low quality of life [16]. Additionally, in a study by Padilla et al., which was conducted among children, AR was present in 66.4% of asthmatic children, and there was a marked association between AR and asthma control [18]. Otherwise, in Vázquez Nava et al.'s study, there was no relation between poor or no asthma control and current AR [17]. Moreover, our findings propose that moderate to severe persistent AR was more prevalent in patients with uncontrolled asthma, followed by partially or controlled patients with asthma.

Allergic rhinitis

For many years, there has been a large body of research supporting the impact of asthma. It is also acknowledged that there has been a principal reduction in quality of life [19]. A study of the national Saudi household survey (Moradi-Lakeh et al.) published in 2013 found a considerable burden of chronic medical problems, including asthma, among Saudis aged 15 years and older. This study found that the self-reported clinical diagnosis of asthma was 4.5%, which is consistent with our findings [20]. Moreover, another study conducted by Jaggi et al. indicated the correlation between asthma and AR [21]. Most often, allergic diseases occur with associated conditions, and AR is no exception to this coexistence. Many studies worldwide have consistently demonstrated that the coexistence of asthma and AR in the same patient is common. The pathophysiology and inflammatory processes of AR and asthma, both of which are IgE-mediated allergies induced by comparable allergens, overlap. Thus, the presence of AR is considered a risk factor for both the incidence and the severity of asthma. The coexistence of AR and asthma can also cause these patients often undergo rounds of consultations with pulmonologists, allergy experts, otolaryngologists, general practitioners, and pediatricians before receiving the appropriate treatment. The concentration of asthma management is primarily on asthma control, with little attention given to the associated rhinitis, which is frequently overlooked. According to Jaggi et al.'s study, the prevalence of AR among patients with asthma in India was as high as 65.24% [21]. Table 4 shows that there are no significant differences between asthma control levels in the prevalence of sneezing, runny nose, and blocked nose over the past 12 months. Additionally, a similar trend was observed regarding the prevalence of itchy-watery eyes and seasonal patterns of nose problems among patients with controlled, partially controlled, and controlled asthma. However, Badran et al.'s study correlated 52.1% of patients with mild clinical manifestations, whereas 47.9% had moderate to severe manifestations [7]. Those with intermittent indications are more likely to experience rhinorrhea and sneezing, whereas those with persistent manifestations are more likely to experience nasal stiffness. Nevertheless, there was a significant correlation between asthma control levels and sensitivity to triggering factors. Patients with controlled or partially controlled asthma were more sensitive to pollen. Patients with asthma who have poorly controlled asthma could not identify specific triggers that lead to the condition. Aside from house dust mites, controlled and partially controlled patients also reported sensitivity to dust and animals, respectively, as the second and third triggers. However, it has been reported that fungi and mold have not been detected. Most patients did not report specific seasons when their symptoms flared up. More patients with partially controlled asthma tested positive for SPT and IgE by 13.6% compared to those with controlled or uncontrolled asthma, and the association was statistically significant (P < 0.001). Nonetheless, the three groups showed no significant difference in the prevalence of eczema (P = 0.159).

Limitations of the study

The study was conducted over a long period of more than one year, and climate changes may cause more symptoms similar to allergy symptoms, which may cause bias. No clinical diagnosis of asthma or allergic diseases contributed to a bias in the results. This study did not include children, which gives a narrower perspective on analyzing children’s data. Participants in this survey were identified based on their self-reports of asthma symptoms and AR symptoms. Additionally, we recommend that a longer survey be conducted over a larger group of individuals with standardized medical intervention to ensure a more accurate diagnosis.

## Conclusions

Asthma and AR are both common respiratory diseases worldwide, and they affect many adults in Saudi Arabia. However, published studies in Saudi Arabia that study asthma with AR are few, concentrated on children only, and insufficient in many aspects, including the prevalence and the effect of AR on patients with asthma and the impact of different risk factors of asthma in asthmatic patients with AR. Thus, this deficiency motivates us to conduct this research to examine these many characteristics to aid a big proportion of society, particularly patients with asthma who have AR. This study suggested that AR was related to more severe asthma and more difficulty controlling asthma. The frequency significantly increased with the severity of asthma. These findings support the need to evaluate rhinitis and analyze treatment choices and strategies for AR in patients with asthma who have AR to enhance asthma control.
